# Supplementary material for: Deglaciation-enhanced mantle CO2 fluxes at Yellowstone imply positive climate feedback
Source: Nat Commun. 2024 Feb 20;15:1526. doi: 10.1038/s41467-024-45890-z (PMC10879189; doi:10.1038/s41467-024-45890-z)
Supplement: Supplementary file 1 — Supplementary Information [file 41467_2024_45890_MOESM1_ESM.pdf]

Supplemental Information for "Deglaciation-enhanced mantle CO_2 fluxes at Yellowstone imply positive climate feedback"

Fiona Clerc[1,2], Mark D. Behn[3], and Brent M. Minchew[4]

1. Previously at: MIT-WHOI Joint Program in Oceanography/Applied Ocean Science & Engineering, Cambridge MA, USA

2. Now at: Lamont-Doherty Earth Observatory, Columbia University, Palisades NY, USA

3. Dept. of Earth and Environmental Sciences, Boston College, Chestnut Hill MA, USA

4. Dept. of Earth, Atmospheric and Planetary Sciences, Massachusetts Institute of Technology, Cambridge MA, USA

*Corresponding author: Fiona Clerc (fclerc@ldeo.columbia.edu)

This file contains:

- Supplementary information text (Sections S1–S5)
- Supplementary figures (Figures S1–S16)

S1. Benchmark against Jull and McKenzie

We benchmarked our numerical model against the semi-analytical model of melt production beneath Iceland of Jull and McKenzie[1], hereafter JM96. Specifically, we replicated their constant mantle potential temperature model, using the same parameters – a viscoelastic half-space of viscosity 8×10^{18} Pa s and shear modulus 0.25×10^{11} Pa, and a parabolic ice sheet extending 180 km in radius and 2 km thick, thinning uniformly over 1000 years.

The rates of pressure change from our numerical model are similar in magnitude to the JM96 solution (compare our Figure S1 with Figure 3 of JM96, noting the different x-axes). During unloading (t = 10–9 ka in JM96; first two panels in Figure S1) the contours of pressure change follow the same pattern. The depth of maximum pressure change after unloading ($t < 9$ ka; bottom panel) is slightly shallower in our model. This may be attributed to the Cartesian load implied by our 2-D model, whereas JM96 employ a radially-symmetric load.

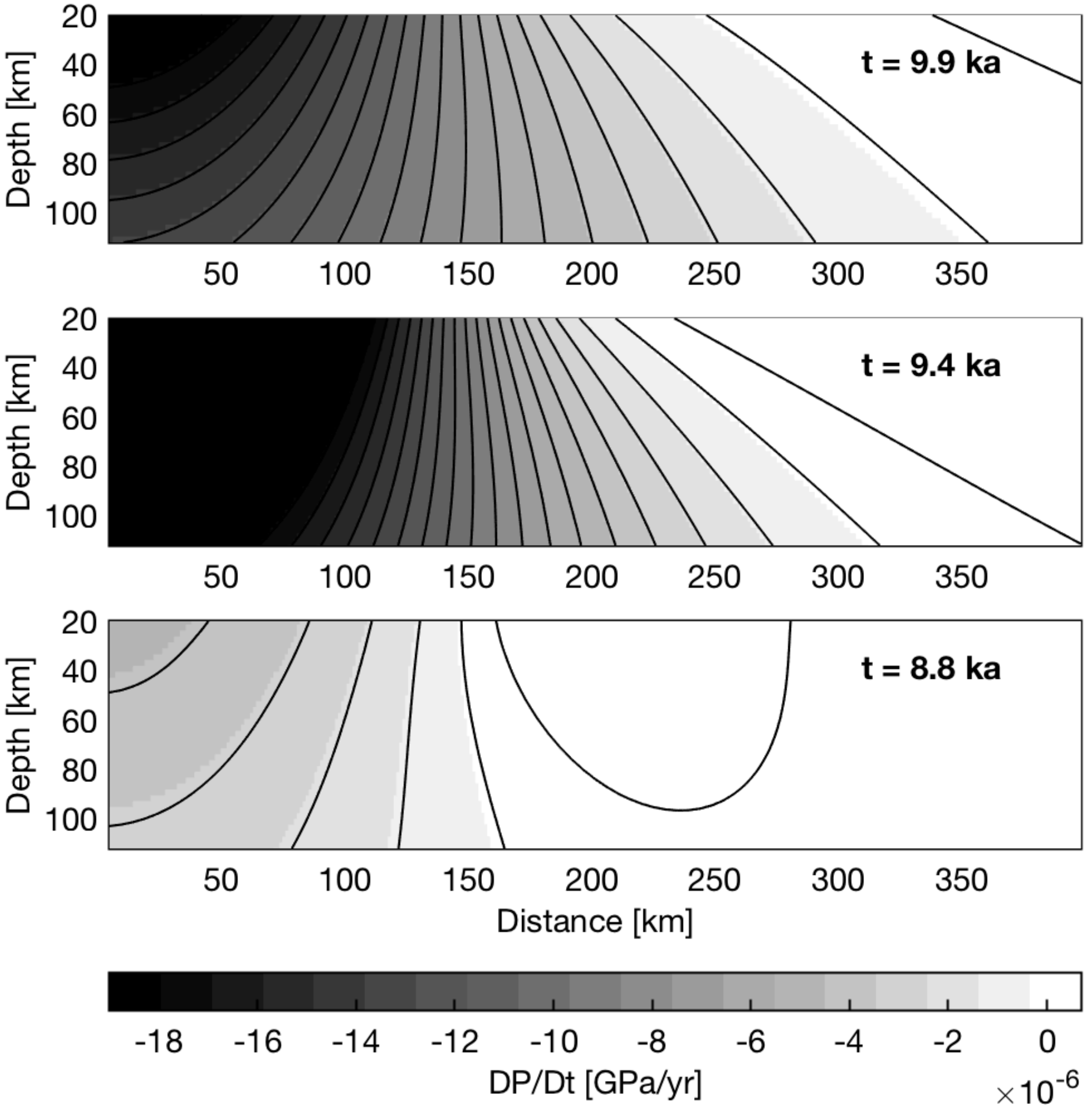

Figure S1: Rates of pressure change, *at different time steps during unloading (10–9 ka). Comparable with figure 3 of JM96.*

We then compared the total melt production rate between models (Figure S2). The melt production rate obtained from our 2–D numerical model should be equivalent to the values JM96 obtain at the ridge axis, i.e., at the x-intercept of their Figure 10a (red stars, Figure S2). JM96 do not specify which solidus they use, but they do limit the region of melting using a 45° triangle

truncated at depths 20-112 km. We obtained similar results with the dry solidus of Katz et al.[2], limited over the same region (compare red lines and stars in Figure S2).

In the primary model presented in the main text, we obtained a higher melt production rate than the model benchmarked against JM96, as we use a different set of assumptions. We performed a step-by-step comparison of the assumptions made in the JM96 model and our primary model, to explore why our estimate is more productive. The main difference arises from the inclusion of enhanced melting from the wings of the melting region in our model (compare red and dashed blue lines in Figure S2). JM96 had limited the width of the triangle to 92 km, while in our model melts are produced out to 300 km from the ridge axis. It is unclear whether (and how rapidly) these peripheral melts would be focused to the ridge axis. The inclusion of the temperature derivative in calculating the rate of melt fraction change (as in ref. [3]) lessens melt production considerably (compare dashed and solid blue lines). Further, the dynamically-consistent plume thermal structure (compare green and blue line) and the inclusion of thermal buoyancy (compare black and green line) also increase melt production.

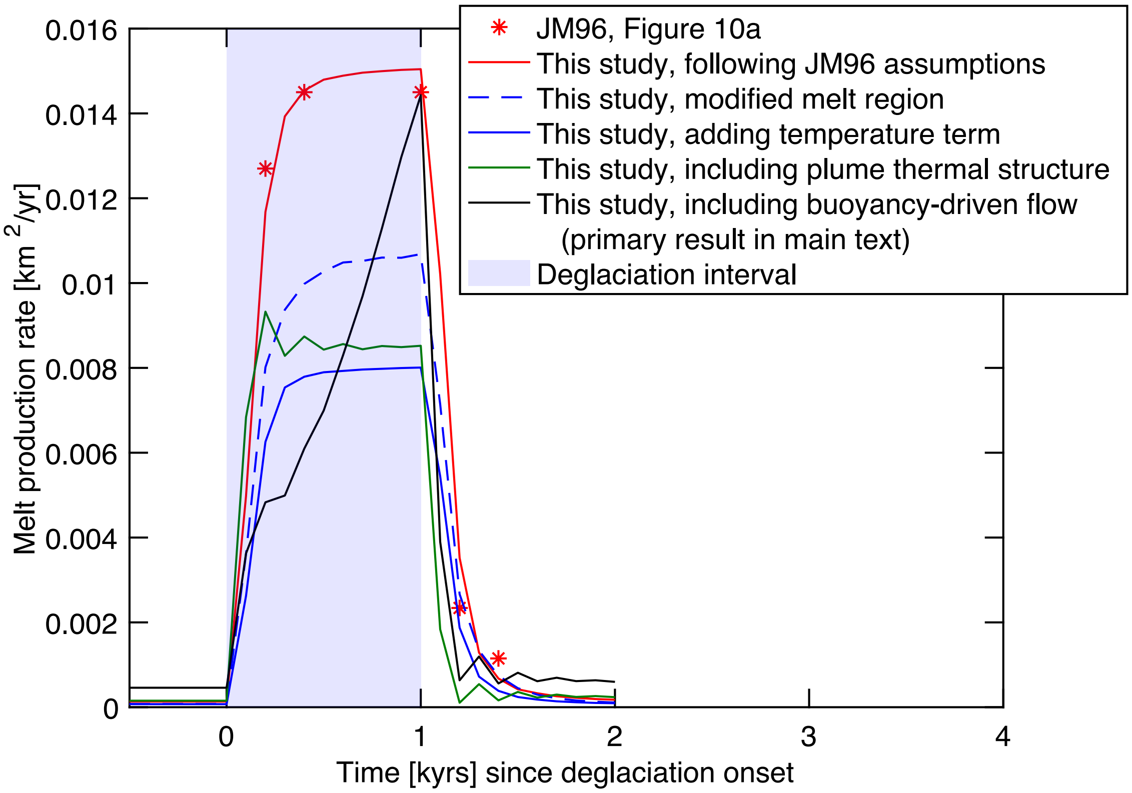

Figure S2: Model runs illustrating step-by-step the effect of modifying assumptions from JM96 *(all under the same glacial forcing). Their results are plotted as red stars. The red line is the most similar/benchmark run, in which the melting region is limited to a truncated triangle extending 92 km off-axis. The dashed blue line shows the effect of using the full melt region predicted by the Katz et al.[2] solidus. The solid blue line shows the effect of including the dependence of melting rate on temperature changes. The green line shows the effect of using the plume thermal structure, but turning thermal buoyancy off. The thick black line shows the dynamically-consistent model with the plume thermal structure and buoyancy-driven flow, as presented in main text.*

We also calculated trace element profiles using the melt fractions and melt production rates from the JM96 benchmark. Despite using a different method (retained batch melting instead

of fractional melting) and partition coefficients, we approximate their reported 15% depletion for the LREE and near 0% for the HREE (Figure S3), when comparing unloading timesteps to background timesteps. While assuming a larger retained melt fraction of 3 wt.% yields the best agreement with JM96 (dashed red line, Figure S3), a smaller value of 1 wt.% (solid lines, Figure S3) better matches observations and is used in the remainder of this study. A comparison of our JM96 benchmark (solid red line) against that of our primary model presented in the main text (solid black line) yields larger depletion of trace elements (~60 %).

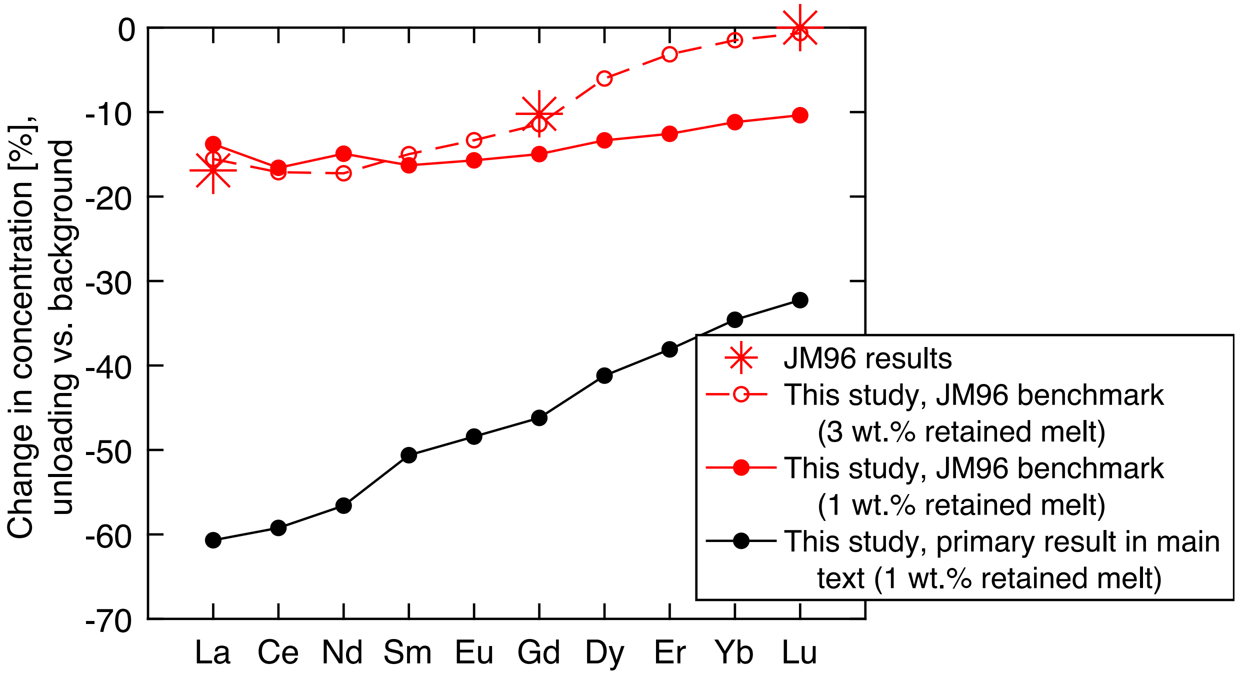

Figure S3: Percent change in trace element concentrations*, comparable with JM96, for an unloading time step (halfway through deglaciation) relative to the background. Our results for the benchmark model (red lines) agree with that of JM96 (red stars). Our results for the primary model (black line) presented in the main text and Figure S14a predict a more important change.*

S2. Iceland sensitivity analysis

S2.1 Effect of ice sheet history

We modeled the effect of the different loading functions used by JM96 (thinning parabola), Eksinchol et al.[4] (viscous gravity current), and this study (retreating parabola). For the models in which the ice sheet retreats inwards, high rates of decompression are initially localized off-axis and then move inwards to the ridge axis. As the mantle is most productive at the axis, the horizontal retreat models predict an increase in total melt production rate through time, while the thinning model from JM96 stays relatively constant during the deglaciation interval (Figure S4a). The viscous gravity current function involves smaller ice volumes, leading to lower melt production rates (green, Figure S4). The total volume of melt produced over the entire deglacial interval scales with the volume of ice lost.

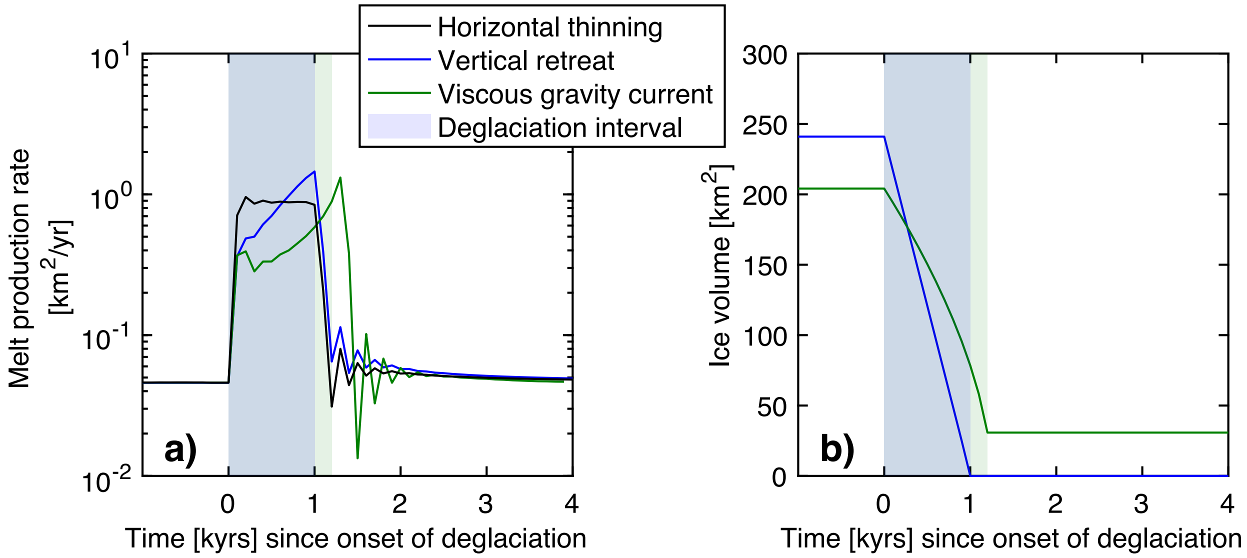

Figure S4: Effect of different loading functions on melt production rate for the Iceland model *(a), including a horizontally thinning parabola as in JM96 (black), a parabola retreating vertically from margins as in main text (blue), and a viscous gravity current (from ref.* [4]*). Corresponding ice volumes in 2-D are plotted in b).*

S2.2 Effect of spreading rate and mantle temperature

The primary Iceland model presented in the main text (blue line in Figure S5) has a spreading rate of 10 mm/yr and a mantle potential temperature of 1300 °C, excluding the excess plume temperature of 175 °C. In the absence of a plume, these parameters yield a steady-state crustal thickness of 7 km. Faster spreading rates of 20 mm/yr (purple line) and warmer mantle temperatures of 1320°C (red line) increase rates of melting prior to and during glacial unloading.

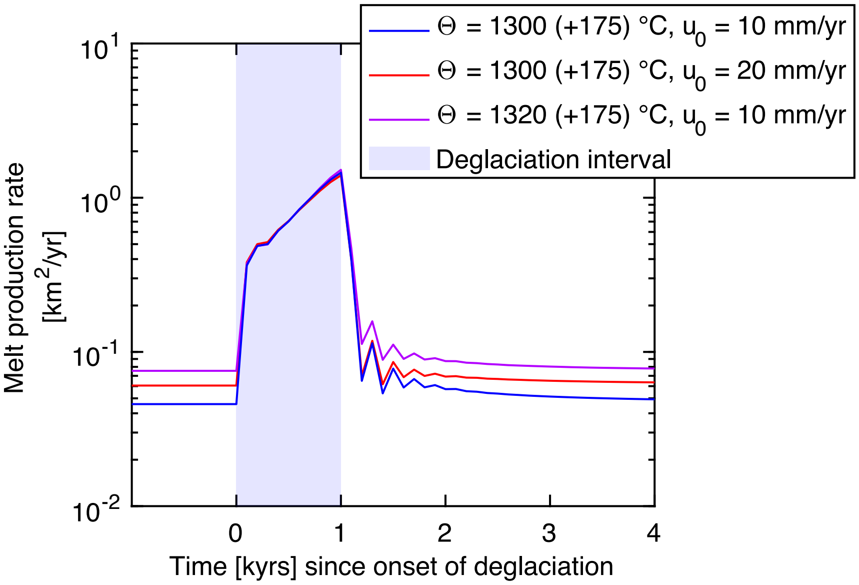

***Figure S5: Effect of increasing temperature and spreading rate, for the Iceland model**. Melt production rates for the case presented in the main text (blue), for a doubled spreading rate (red), and for a raised mantle potential temperature (purple).*

S2.3 Effect of pooling width

Prior to glacial loading, our model predicts that melt production occurs at distances up to 150 km from the ridge axis (positive melt fraction rates in Figure 2b). Assuming all melt is focused to the ridge axis, we predict the production of a 120 km-thick crust – larger than the 20–40 km observed seismically[5,6]. However, melts produced far from the ridge axis may refreeze at the base of the lithosphere during melt migration, thereby not contributing to on-axis crustal

production (e.g., ref. [7–9]). We find that by limiting the pooling width to 30 km in our model, we obtain a 39 km-thick crust (orange circle in Figure S6a), matching seismic observations beneath Iceland[5,6]. This pooling width is similar to the ~25 km estimate[9] derived from a global analysis of mid-ocean ridge basalt geochemistry and crustal thickness and is significantly narrower than the full width of the melt production region.

Imposing a pooling width also limits the flux of CO_2 (Figure S6b), because we assume only those melts that reach the ridge axis will degas their CO_2 to the atmosphere. By contrast, CO_2 transported by melts that refreeze in the lithosphere may partially exsolve. Because we assume off-axis melts refreeze at the base of the lithosphere (defined as the 1100ºC isotherm), the pressure-dependence of CO_2 solubility implies that the contribution of off-axis melts to the CO_2 flux is only important under high mantle source CO_2 concentrations (solid lines in Figure S6b). In other words, for low source concentrations (~150 ppm) melts that refreeze at depth will not be saturated in CO_2 and thus will minimally contribute to the total CO_2 degassing flux (dashed lines in Figure S6b).

Finally, we find that the enhancement in the rates of melting and CO_2 production relative to the background rate, increases with the pooling width (compare black and orange lines in Figure S6). This illustrates how glacial unloading extends the region over which melts are

produced (see Figures 2b&d).

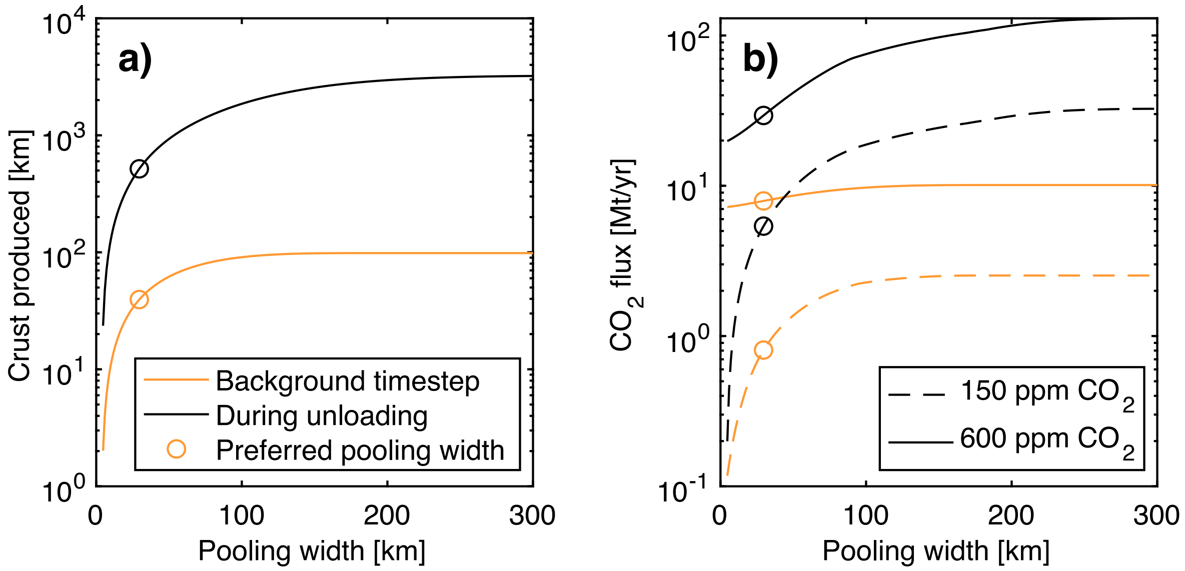

Figure S6: Effect of pooling width *on a) crustal production and b) CO_2 production rate, for the Iceland model. Orange lines represent background production rates associated only with the plume and ridge before glacial loading. Black lines show deglacially-enhanced rates, midway through the deglaciation. Open circles show the preferred 30-km pooling width required to produce the seismically observed crustal thickness. CO_2 fluxes assume a mantle source CO_2 concentrations of 150 ppm (dashed) and 600 ppm (solid), respectively.*

S3 Yellowstone sensitivity analysis

S3.1 Effect of mantle temperature

The primary model for Yellowstone presented in the main text has a background mantle potential temperature 1320°C, plus an excess plume temperature of 80°C (i.e. 1400°C mantle potential temperature at the plume center). These parameters yield a background mantle melt production rate of 0.022 km^3/yr (black lines in Figures S7a and 3c), within the range of estimated crustal emplacement rates of basaltic mantle melts[10,11]. We explore the effect of increasing the

background mantle temperature to 1340°C and 1360°C, yielding background mantle melt production rates of 0.056 km^3/yr (as in ref. [12]) and 0.15 km^3/yr (as in ref. [13]), respectively. To calculate CO_2 fluxes (Figures S7b and 3e), we use mantle source CO_2 concentrations of 150–600 ppm in the 1320°C case, 190–675 ppm in the 1340°C case, and 120–375 ppm in the 1360°C case. These concentrations yield background CO_2 fluxes of 1.7–7.3 Mt/yr, consistent with modern constraints[10,14].

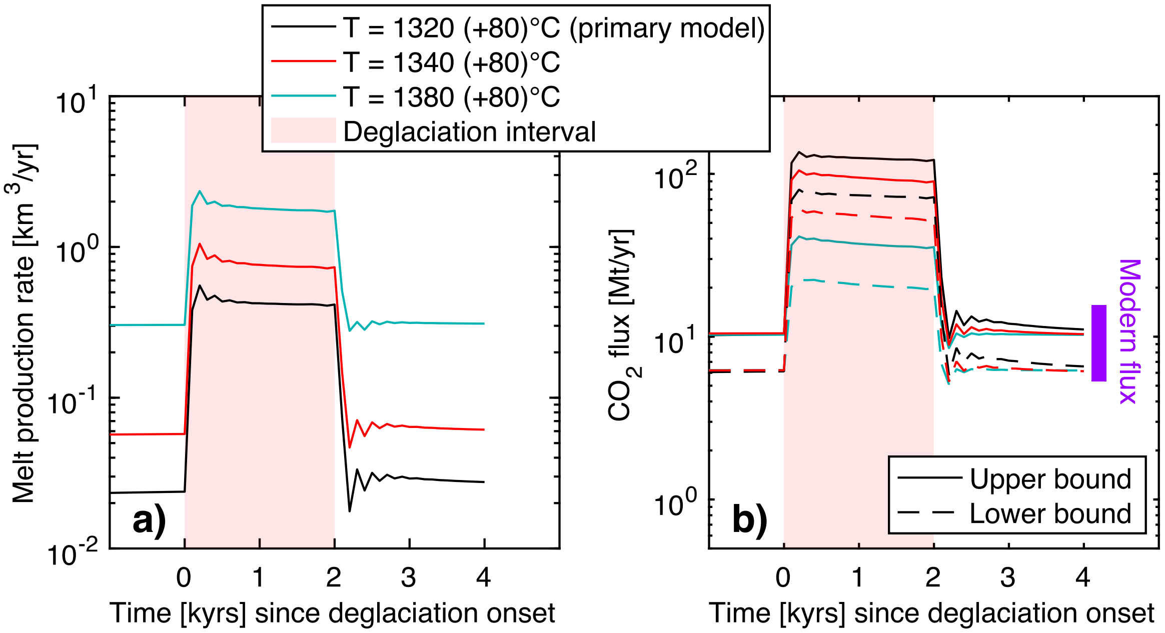

Figure S7: Effect of mantle temperature, *on melt production rates a) and CO_2 flux b), for the Yellowstone model. Black lines are results presented in the main text. Source mantle CO_2 concentrations in b) are varied to match the modern flux (purple bars).*

S3.2 Yellowstone without plume

While it is established that there is additional melting in the upper mantle beneath Yellowstone, the presence of a mantle plume extending to depths of 600 km or greater remains controversial[15]. We perform a run in which we remove the plume tail, to understand its effect on our model. To do so, we artificially lower the temperature of the mantle at greater depths (>150

km), such that only an upper mantle thermal anomaly remains. Removing the plume tail and reducing the influx of material lessens upwelling from the lower mantle. We find the rates of pressure change in the melting region during unloading are similar, implying the presence/absence of the plume tail itself does not significantly affect our results (they are instead controlled primarily by the viscosity of the upper mantle in the melting region, and overlying lithosphere). As the thermal anomaly at the base of the lithosphere was originally set by the plume, this test is not equivalent to explicitly modeling another mechanism (e.g., edge-driven subduction from the slab).

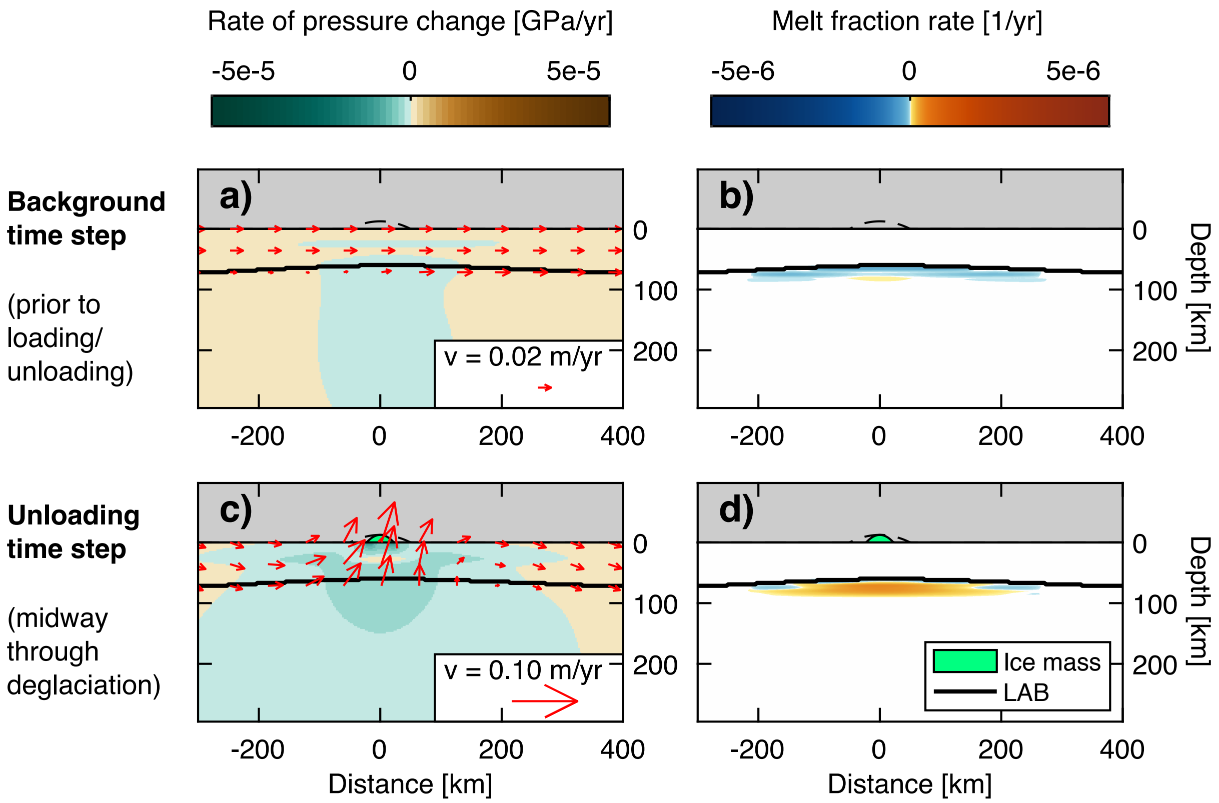

Figure S8: ***Effect of the deglaciation of the Yellowstone ice cap*****, *in the absence of a plume*** *(green parabola) on rates of pressure change (a,c, teal-brown colors) and rates of melt fraction change (b,d, blue-orange colors). The top row shows a model time step prior to any glacial loading/unloading, while the bottom row shows a time step halfway through the deglaciation*

(1000 years following its onset). Red arrows show mantle flow, the thick black line is the LAB (T=1300°C).

S3.3 Effect of loading function

Given the thickness of the lithosphere and the great depth of melting beneath Yellowstone, the pressure changes due to unloading are distributed throughout the melting zone. As a result, the style of ice cap retreat is unimportant (Figure S9a). Upon radial integration, ice caps which are thinned horizontally yield nearly identical rates of melt production compared to ice caps that retreat vertically, upon radial integration. If the ice cap retreats more slowly, the melt production rate decreases proportionately but the total volume of extra melt produced is unchanged. Our estimates of the total extra melt and CO_2 are produced by the end of the deglaciation does not depend on the manner in which the ice cap retreats, given a constant initial ice volume.

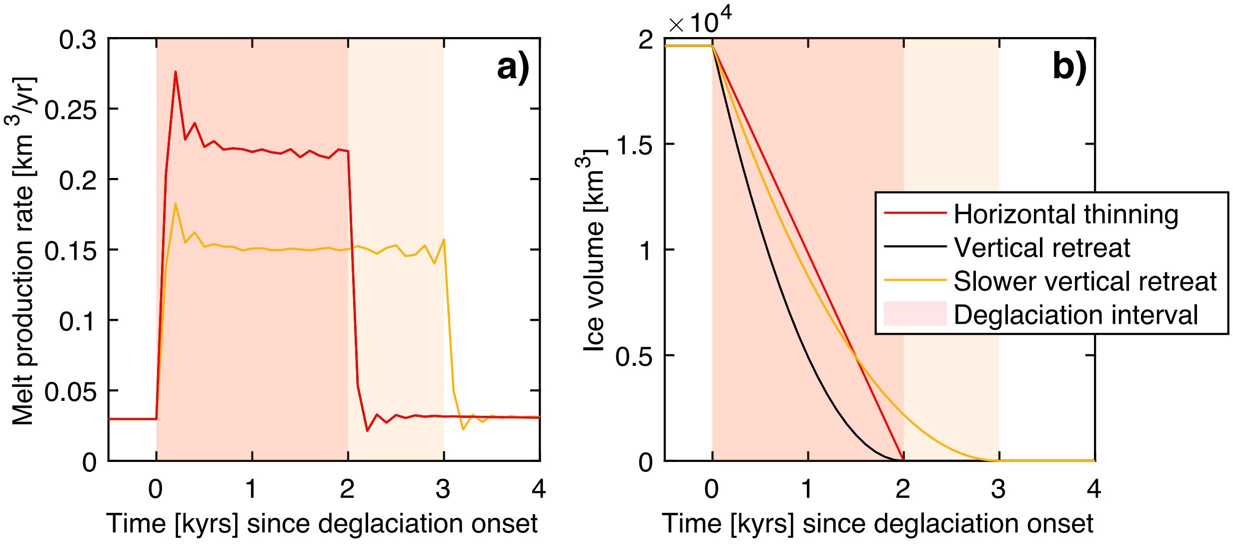

***Figure S9: Effect of different loading functions on melt production rate** (a), including a horizontally thinning parabola (red), a parabola retreating vertically from margins as in main*

text (black), and a slower deglaciation lasting 3000 years (orange). Corresponding radially-integrated ice volumes are plotted in b).

S3.4 Effect of lithospheric rheology

One of the primary differences between Yellowstone and Iceland is the thickness of the lithosphere. We used semi-analytical models (as in JM96) to examine the influence of lithosphere rheology on enhanced mantle melting due to deglaciation, by varying the shear modulus and lithosphere viscosity in a viscoelastic half-space at a point located 70 km beneath the load center (Figure 5a). We find that the total change in pressure following a deglaciation (which is a proxy for the cumulative volume of melt produced) reaches steady-state values in the limits of softer/more rigid lithosphere (upper/lower bounds, respectively). To examine the effect of lithospheric rheology in our numerical models (while maintaining the same asthenospheric viscosity profiles), we run additional simulations with shear moduli of 0.3 and 1000 GPa (Figures S10a, S11a). This range bounds the 10 GPa used in the primary models presented in the main text (solid black lines in Figure S10, S11). Under smaller shear moduli, decompression is more important directly underneath the load (solid red line, Figure S10) at the relevant depths of melt generation (70–90 km). However, this effect is offset by increasing compression outwards from the load center for small G (dash-dotted lines), implying that the total melt produced throughout the domain is relatively insensitive to the elastic properties of the lithosphere (Figure S11a).

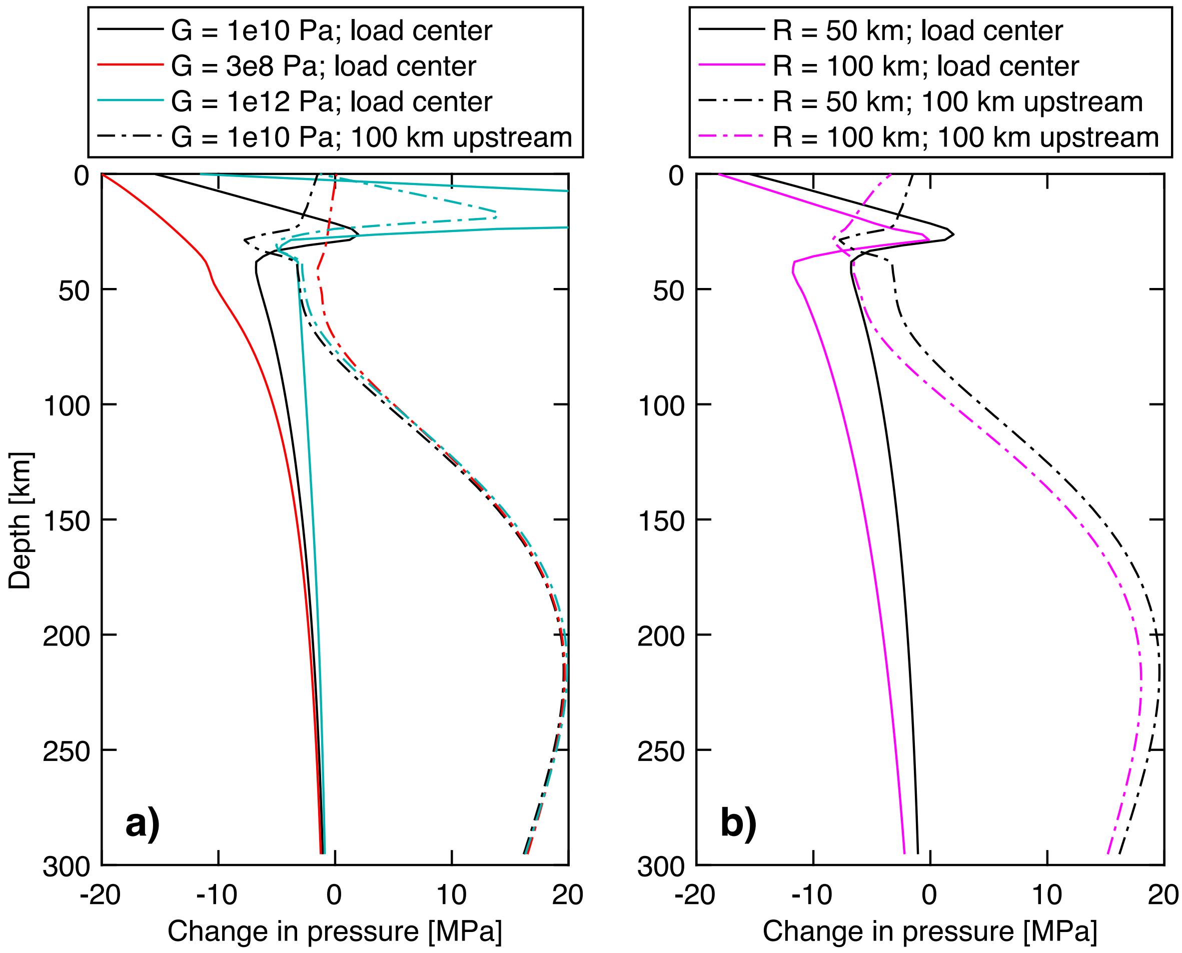

Figure S10: Depth dependence of pressure change, following Yellowstone deglaciation*. Solid lines are profiles directly beneath the load, dash-dotted lines are profiles located 100 km upstream (eastward) of the load. Total change in pressure is dependent on: a) shear modulus (G) and b) load radius (R). These numerical results are comparable to semi-analytical parameter space in Figure 5.*

We also note that the pressure change has an exponential dependence on depth over the load radius (Figures 5b, S10b, S11b). The smaller load and deeper locus of melting beneath Yellowstone explains the more muted response (relative to Iceland). This scaling further

motivates investigations into the effect of deglaciation on mantle melting beneath WAIS, given its large horizontal extent.

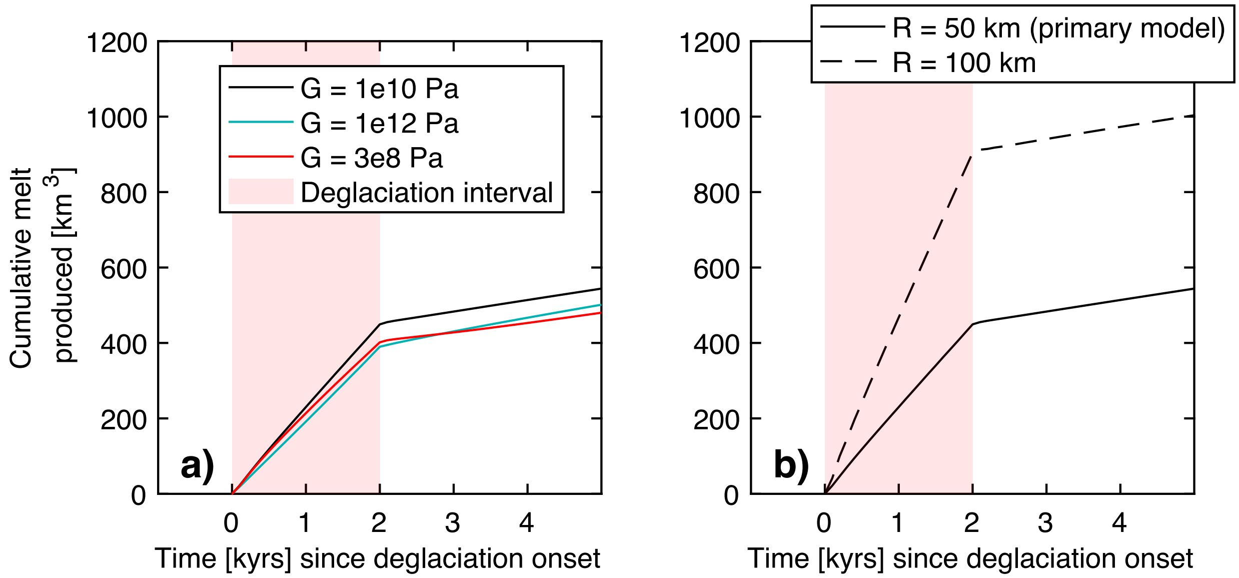

Figure S11: Sensitivity of cumulative melt produced to shear modulus and load radius following Yellowstone deglaciation*. The primary model from the main text is plotted as black solid lines. a) Shear modulus (G) has limited influence on cumulative melt produced. b) Load radius (R) exerts a primary control on melt produced. These numerical results are comparable to semi-analytical parameter space in Figure 5.*

S3.5 Comparison between 2-D and 3-D

We assume a 2-D Cartesian geometry in which the applied loads extend infinitely out of the page. Reconstructions of the Yellowstone ice cap indicate that it had a finite depth or "long axis" of ~150 km, in the direction perpendicular to the plume track/plate motions. To test the sensitivity of our results to these 3–D effects, we performed a 3–D model in which we imposed an elliptical ice load with long axes ("L" in Figure S12) of 150 km, 800 km, and beyond the in-plane domain width (3–D "Cartesian" in Figure S12). Within a slice corresponding to the 2-D

models ("center plane"), we find that using a load with a 150-km long axis reduces the melt production rate by ~70%, relative to the Cartesian case (Figure S12a). We also compare the volumetric melt production rate obtained from the 3-D models (dashed lines in Figure S12b) to the value obtained from radially-integrating melt production rates in map view along the center plane, as done in the 2-D models (solid lines in Figure S12b). Using the 3-D melt production rate instead of the radially-integrated 2-D values increases the estimated melt production rate by ~30%. In combination, these 3-D effects imply the melt production rates from the 2-D models are not obviously biased, and therefore capture first-order behavior (subject to uncertainties in deglacial history).

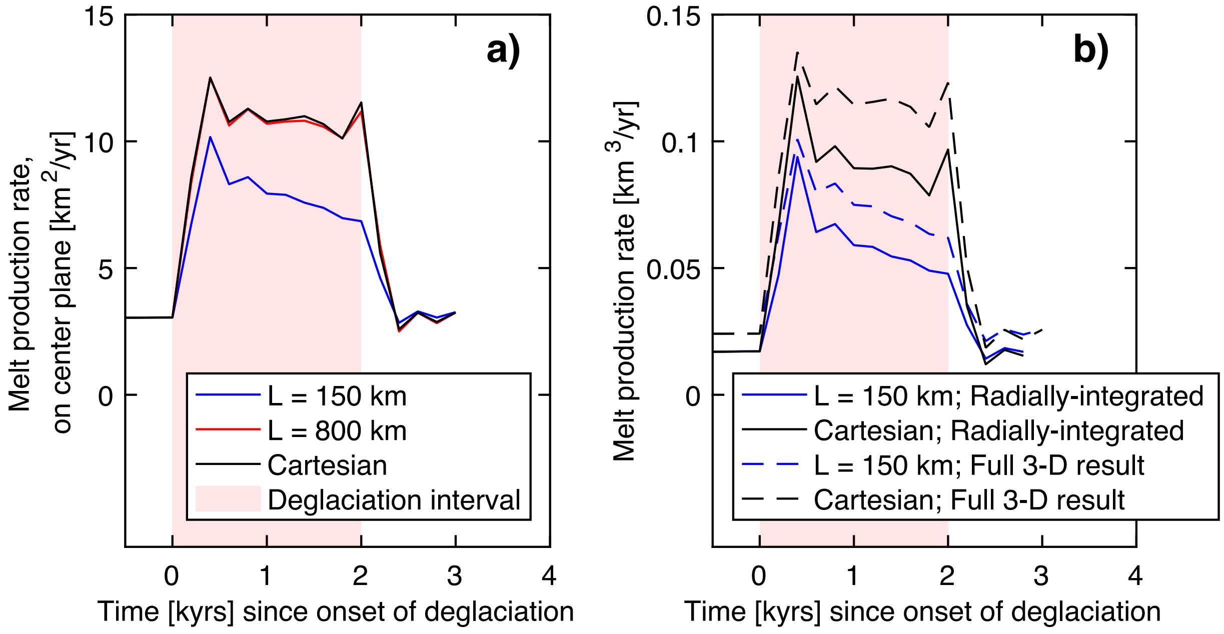

Figure S12: Influence of Cartesian 2-D approximations, examined in 3-D models. *a) Melt production rates in center plane analogous to the 2-D models, for load widths (L) of 150 km (blue line), 800 km (red line), and larger than the domain (black line). b) Volumetric melt production rates from full 3-D model (dashed lines) compared with radially-integrated values from center plane, as done in 2-D models (solid lines).*

S3.6 Effect of pooling width

In the Yellowstone model, we benchmarked melt production rates against estimated rates of basalt emplaced underneath the caldera (Werner & Brantley, 2003; Hildreth, 1991). As the areal extent of the caldera and underlying magmatic system is broad, we do not impose a pooling width (in contrast to the Iceland model in which all melt was assumed to be focused to a narrow neo-volcanic zone). We calculate the cumulative melt production with increasing distance from the plume head (Figure S13a), and find that most of the melt is generated within a 100 km radius.

Implementing a pooling width would imply that the melts generated near the caldera exterior may not reach the shallow magma chamber, instead refreezing at depth. These melts would contribute smaller amounts of CO_2 than those emplaced at 14 km, near the base of the magma chamber. However, this effect is minimal (Figure S13b), as these low-degree melts contain high concentrations of CO_2, well above the concentration of CO_2 soluble in the melts at depths of 14–70 km. Overall, we conclude that the uncertainties in mantle CO_2 flux associated with pooling width are small relative to uncertainties associated with the background rate of mantle melting beneath Yellowstone (see Section S3.1).

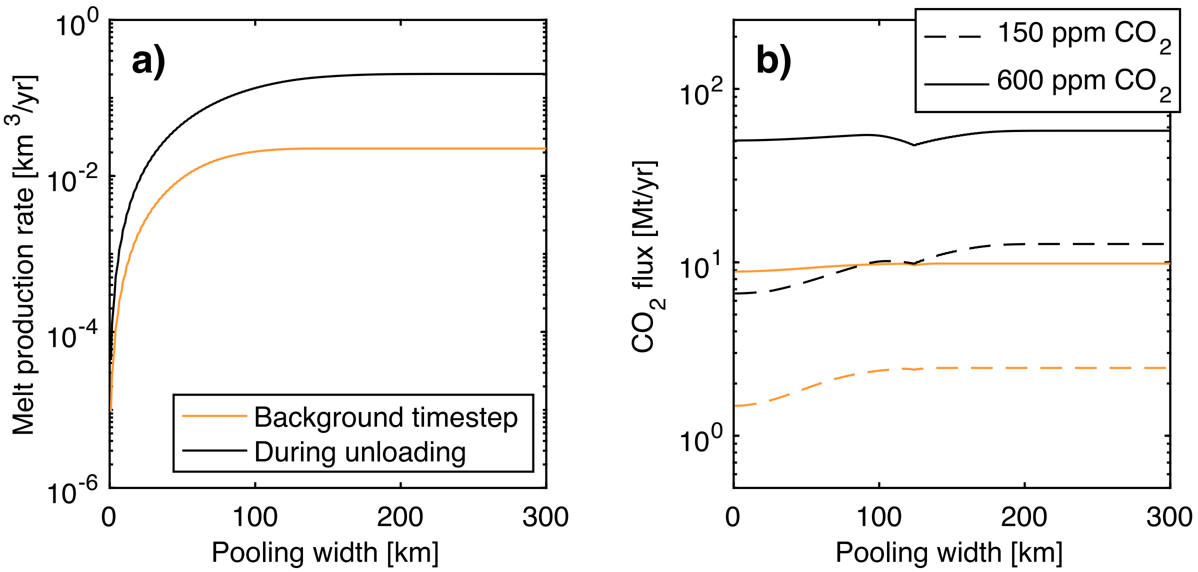

Figure S13: Effect of pooling width *on a) melt and b) CO_2 production rates during Yellowstone deglaciation. Orange lines represent background rates, prior to any glacial loading. Black lines show deglacially-enhanced rates, midway through the deglaciation. CO_2 fluxes assuming mantle source CO_2 concentrations of 150 ppm and 600 ppm are plotted as dashed and solid lines, respectively.*

S4. Trace elements

We calculate trace element profiles for both Iceland and Yellowstone (Figure S14). These profiles compare well with erupted basalts, which provides support for the CO_2 calculations in the main text. Our modeled Iceland profiles produce a sufficiently large percent change between background and unloading time step (Figure S14a). For Yellowstone, trace element compositions both before and during unloading are within the range of the data (Figure S14b). None of these basalts were dated to the Bull Lake deglaciation (140-150 ka). The percent change predicted by the model (~30%) may be too small to be detected, even if basalts dated to the deglaciation were

found. We also show the sensitivity of our results to the choice of retained melt fraction (Figure S15); a value of 1 wt. % best fits the data.

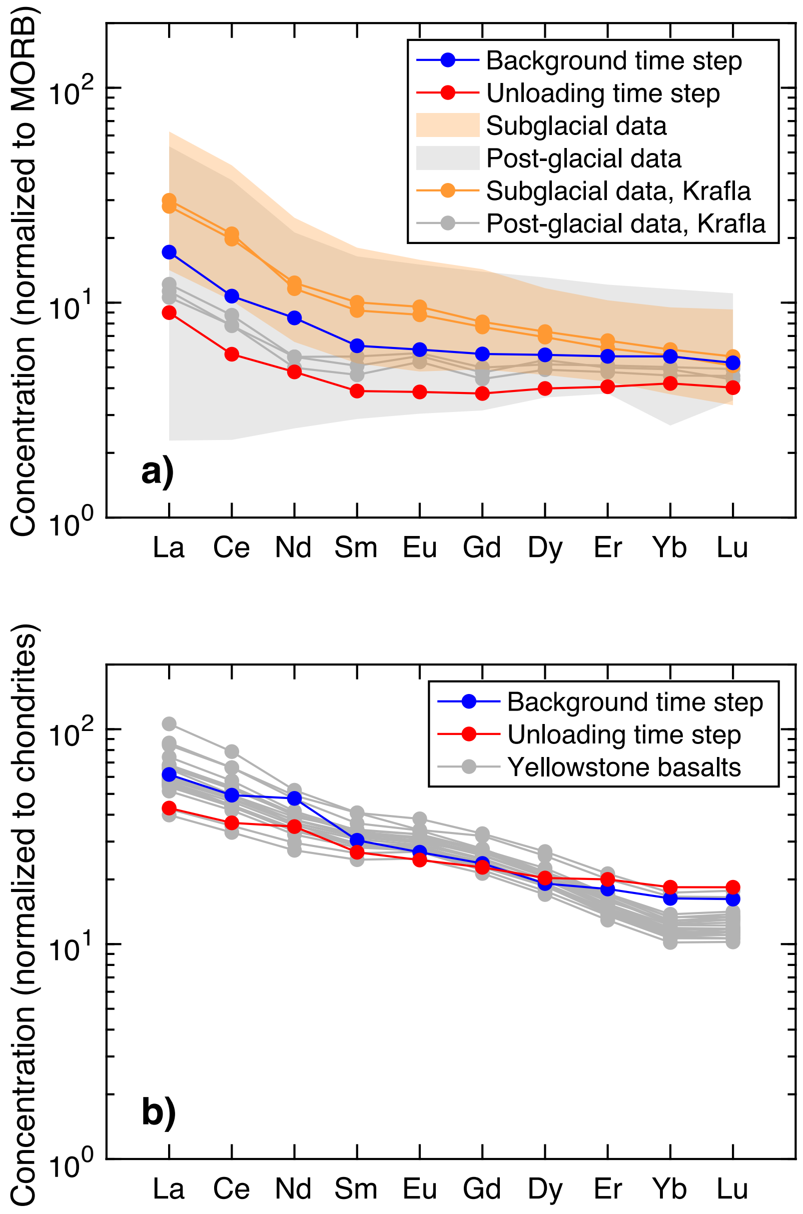

Figure S14: Pooled trace element concentrations before and during unloading, pooled over model domain. *a) Iceland model results compared to data from Maclennan et al.*[16] *Trace element depletion is most apparent when comparing subglacial (74 –12.5 ka) to postglacial (12.5 – 10 ka) data from the same volcano (for example, Krafla is a volcano located in Iceland's*

northern volcanic rift zone). b) Yellowstone model results compared to data from Bennett[17]. *The Iceland and Yellowstone data are normalized to MORB and chondrites, respectively (using ref.* [18]*), for ease of comparison with the original datasets.*

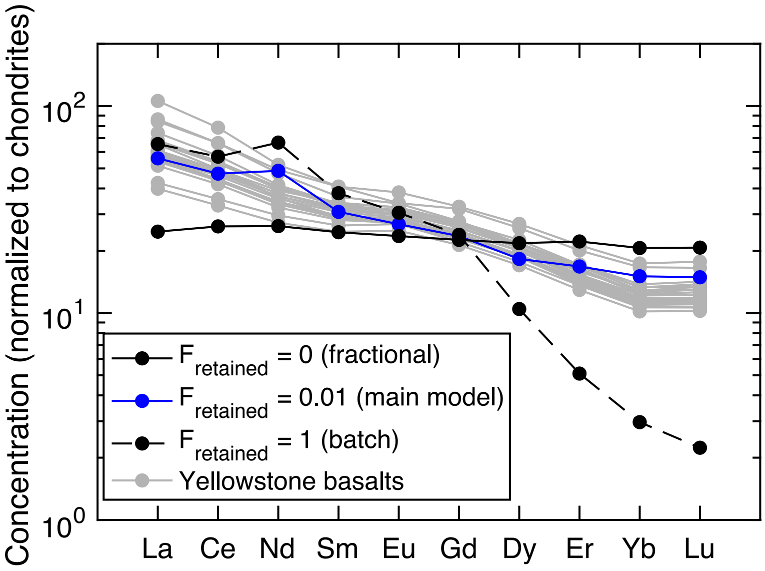

Figure S15: Influence of retained melt fraction ($F_{retained}$) on trace element concentrations in Yellowstone*, for 1-D melt column during background time step. Fractional melting is approximated under no melt retained (solid black line), batch melting under all melt retained (dashed black line). Blue line shows model used in main text (1 wt. % retained melt).*

S5. Mantle CO_2 depletion

In calculating the concentration of CO_2 in the melt, we assume that the concentration of CO_2 in the mantle (c_s) remains in steady-state. In reality, additional melting due to deglaciation may deplete the mantle and reduce the CO_2 flux estimate. We explore the magnitude of this effect in a 1-D profile near the center of the plume (Figure S15). We use the modal fractional/batch melting end-members and assume a partition coefficient of 5.5×10^{-3} (ref. [19]). These assumptions provide easily differentiable expressions for the rate of change in c_s (Figure S16a,b), consisting of an advective term (magenta lines) and change in c_s due to melting (black

lines). The difference between these terms, integrated through time, represents the CO_2 extracted from the mantle by melts (Figure S16c). Assuming a mantle source concentration of 100 ppm, under steady-state ("background time step") the mantle loses <1 ppm after 1000 years, at depths of 85–90 km. During unloading, an additional 1–2 ppm is lost on average (difference between red and blue lines in Figure S16c. Thus, we estimate the transient depletion in mantle CO_2 may reduce our deglacial flux estimates by 1–2%. This effect is small compared to uncertainties in the background CO_2 flux (e.g. [14]).

Finally, the CO_2 is sourced from a 3–5-km deep region at the base of the melting domain. This region is replenished by upwelling mantle at a rate of ~3–5 cm/yr. Estimates of upwelling rates range from 3 cm/yr (our model) to 5 cm/yr (ref. [20]), where the difference can be explained by the omission of chemical buoyancy in our model. We expect that much of this region (3–5 km) would be replaced by new mantle following a 100-kyr glacial cycle – implying that an immediately preceding deglaciation may influence deglacial CO_2 fluxes, but that these effects are not cumulative over multiple preceding glaciations.

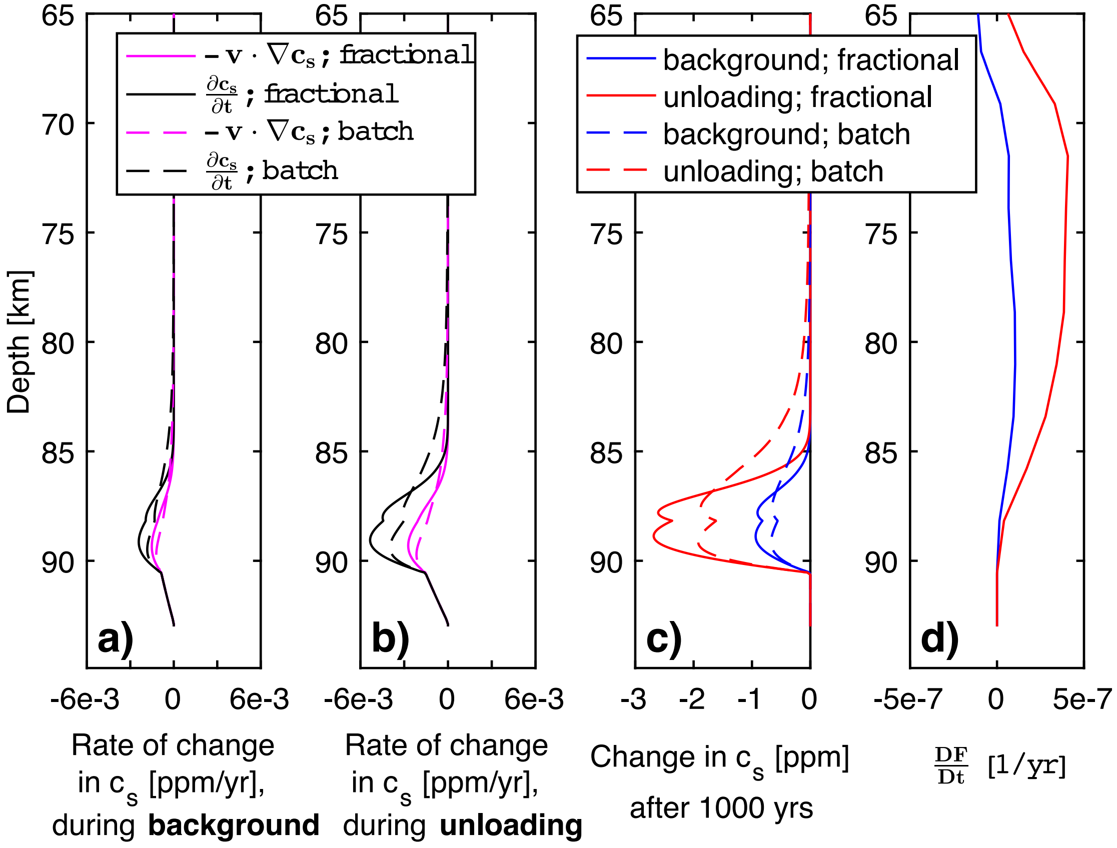

***Figure S16: Concentration of CO_2 remaining in mantle residue (c_s) in a 1-D vertical melt column**, under modal fractional (solid lines) and batch (dashed lines) melting. Rate of change in c_s during a background time step (a) and unloading time step (b), due to advection (magenta lines) and loss through melting (black). c) Total change in c_s after 1000 years, for background (blue lines) and unloading time steps (red). d) Melt production rate for both time steps.*

Supplementary References

1. Jull, M. & McKenzie, D. The effect of deglaciation on mantle melting beneath Iceland. *J. Geophys. Res. Solid Earth* (1996).
2. Katz, R. F., Spiegelman, M. & Langmuir, C. H. A new parameterization of hydrous mantle melting. *Geochemistry, Geophys. Geosystems* (2003). doi:10.1029/2002GC000433
3. Dannberg, J. & Heister, T. Compressible magma/mantle dynamics: 3-D, adaptive simulations in ASPECT. *Geophys. J. Int.* (2016). doi:10.1093/gji/ggw329
4. Eksinchol, I., Rudge, J. F. & Maclennan, J. Rate of Melt Ascent Beneath Iceland From the Magmatic Response to Deglaciation. *Geochemistry, Geophys. Geosystems* (2019). doi:10.1029/2019GC008222
5. Darbyshire, F. A., White, R. S. & Priestley, K. F. Structure of the crust and uppermost mantle of Iceland from a combined seismic and gravity study. *Earth Planet. Sci. Lett.* (2000). doi:10.1016/S0012-821X(00)00206-5
6. Jenkins, J. *et al.* Crustal formation on a spreading ridge above a mantle plume: receiver function imaging of the Icelandic crust. *J. Geophys. Res. Solid Earth* **123**, 5190–5208 (2018).
7. Hebert, L. B. & Montési, L. G. J. Generation of permeability barriers during melt extraction at mid-ocean ridges. *Geochemistry, Geophys. Geosystems* **11**, (2010).
8. Montési, L. G. J., Behn, M. D., Hebert, L. B., Lin, J. & Barry, J. L. Controls on melt migration and extraction at the ultraslow Southwest Indian Ridge 10–16 E. *J. Geophys. Res. Solid Earth* **116**, (2011).
9. Behn, M. D. & Grove, T. L. Melting systematics in mid-ocean ridge basalts: Application of a plagioclase-spinel melting model to global variations in major element chemistry and crustal thickness. *J. Geophys. Res. Solid Earth* **120**, 4863–4886 (2015).

10. Werner, C. & Brantley, S. CO2 emissions from the Yellowstone volcanic system. *Geochemistry, Geophys. Geosystems* (2003). doi:10.1029/2002GC000473

11. Hildreth, W., Halliday, A. N. & Christiansen, R. L. Isotopic and chemical evidence concerning the genesis and contamination of basaltic and rhyolitic magma beneath the yellowstone plateau volcanic field. *J. Petrol.* **32**, 63–138 (1991).

12. McMillan, N., Larson, P., Fairley, J., Mulvaney-Norris, J. & Lindsey, C. Direct measurement of advective heat flux from several Yellowstone hot springs, Wyoming, USA. *Geosphere* (2018). doi:10.1130/GES01598.1

13. Hurwitz, S. & Lowenstern, J. B. Dynamics of the Yellowstone hydrothermal system. *Reviews of Geophysics* (2014). doi:10.1002/2014RG000452

14. Rahilly, K. E. & Fischer, T. P. Total diffuse CO2 flux from Yellowstone caldera incorporating high CO2 emissions from cold degassing sites. *J. Volcanol. Geotherm. Res.* **419**, 107383 (2021).

15. Christiansen, R. L., Foulger, G. R. & Evans, J. R. Upper-mantle origin of the Yellowstone hotspot. *Bull. Geol. Soc. Am.* (2002). doi:10.1130/0016-7606(2002)114<1245:UMOOTY>2.0.CO;2

16. Maclennan, J., Jull, M., McKenzie, D., Slater, L. & Grönvold, K. The link between volcanism and deglaciation in iceland. *Geochemistry, Geophys. Geosystems* (2002). doi:10.1029/2001GC000282

17. Bennett, K. M. Petrogenesis of Pleistocene basalts in the Norris-Mammoth Corridor , Yellowstone National Park. 131 (2006).

18. Sun, S. -s. & McDonough, W. F. Chemical and isotopic systematics of oceanic basalts: implications for mantle composition and processes. *Geol. Soc. London, Spec. Publ.* **42**, 313–345 (1989).

19. Rosenthal, A., Hauri, E. H. & Hirschmann, M. M. Experimental determination of C, F, and H partitioning between mantle minerals and carbonated basalt, CO2/Ba and CO2/Nb systematics of partial melting, and the CO2contents of basaltic source regions. *Earth Planet. Sci. Lett.* **412**, 77–87 (2015).

20. Steinberger, B., Nelson, P. L., Grand, S. P. & Wang, W. Yellowstone Plume Conduit Tilt Caused by Large-Scale Mantle Flow. *Geochemistry, Geophys. Geosystems* **20**, 5896–5912 (2019).
